# Retroperitoneal Ganglioneuroma

**Published:** 2013-01-01

**Authors:** Jitendra Singh, Vinod Kr Priyadarshi, Praveen Kumar Pandey, Mukesh Kr Vijay, Dilip Kumar Pal, Anup Kundu

**Affiliations:** Department of Urology, Institute of Postgraduate Medical Education and Research, Kolkata. India.

A 10-year boy presented with a painless swelling in the left upper abdomen which gradually increased in size. Physical examination revealed a non tender, smooth, firm and an irregular mass in left hypochondrium extending to left lumbar region. On ultrasound, a hypoechoic lesion (14cm x10cm) in left suprarenal region was noted. Computed tomography (CT) revealed a large (18cm x 12cm) lobulated hypodense lesion with linear hyperdense enhancing areas in the left suprarenal region (Fig. 1). The lesion displaced the stomach and pancreas anterio-superiorly, the left kidney posterio-inferiorly and the spleen laterally. The fat planes surrounding these structures were intact. There was stretching of the splenic and left renal veins. Medially, the mass was seen extending anterior to the abdominal aorta. There was anterio-lateral displacement of the portal vein and inferior vena cava while the bowel loops were displaced inferiorly. 

**Figure:**
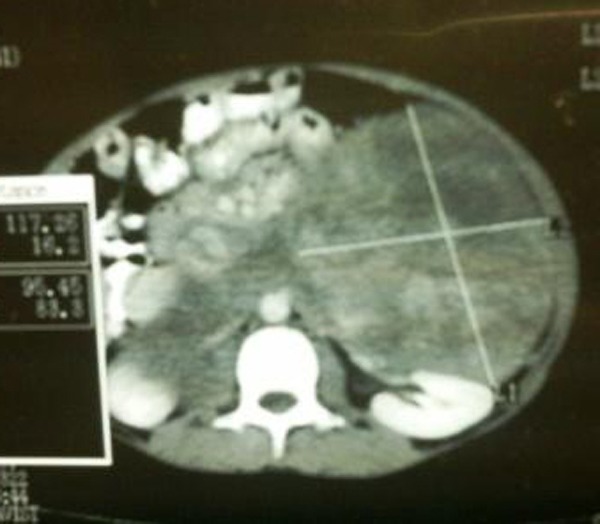
Figure 1: CT scan abdomen.

Serum electrolyte and hormonal studies confirmed the non functional nature. On exploration, the mass was found arising from left suprarenal region. There was no evidence of invasion of the adjacent organs. The resected specimen was pale yellow in color with firm to hard consistency (Fig. 2). The histological examination of the mass revealed a mixture of immature and mature ganglion fibers with the neuronal cells, suggestive of ganglioneuroma. There was no evidence of malignancy. Patient was discharged on post operative day-15 and was kept on 3 monthly follow up with serum electrolyte, serum cortisol and ultrasound. 

**Figure:**
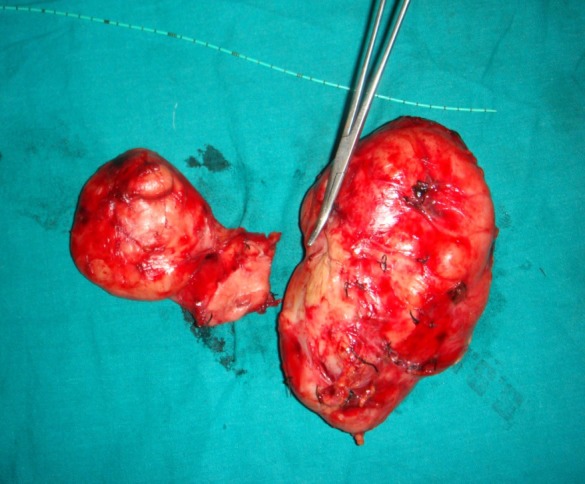
Figure 2: Resected specimen.

## DISCUSSION

Ganglioneuroma (GN) is a rare benign tumor. They may occur spontaneously or during the therapy for neuroblastoma with either chemotherapy or radiation therapy [1]. GNs are mostly sporadic with familial predisposition. Association with Turner’s syndrome and multiple endocrine neoplasia II has been reported [2]. It is most commonly found is posterior mediastinum and the retroperitoneum [2]. The reported incidence of GN is one per million population [3]. As a primary retroperitoneal tumor, it constitutes only a small percentage of 0.72 to 1.6% [3].

Retroperitoneal GNs are usually nonfunctioning and asymptomatic until they reach large sizes, when they cause symptoms due to local expansion and compression of adjacent structures [4]. Ganglioneuroma may release catecholaminergic peptides, which can produce hypertensive crisis during the surgery [4]. 

Preoperative diagnosis of retroperitoneal ganglioneuroma is often difficult and is usually based on histopathological findings after surgical excision of the tumor [4, 5]. CT is the most commonly used imaging modality for assessment because it reveals the extent of the tumor, organ of origin, regional invasion, vascular encasement, adenopathy, and calcification [5]. Interestingly, GNs tend to partially or completely surround major blood vessels, with little or no compromise of the lumen [6]. 

Pathologically, GNs should be differentiated from ganglioneuroblastoma, which have a ganglioneuromatous component of more than 5%; the ganglioneuromatous component of GN is minimal [7]. 

Treatment of GN consists of complete surgical removal of the tumor, when possible [8]. Preoperative or postoperative chemotherapy or radiotherapy has no value, unless the GN is associated with ganglioneuroblastoma changes [9]. The possibility of slow progression and late recurrence, rare malignant transformation has been reported; therefore, long-term periodic radiologic surveillance is necessary [2, 8].

## Footnotes

**Source of Support:** Nil

**Conflict of Interest:** None declared
